# A Randomized Controlled Cluster Trial of an Obesity Prevention Program for Children with Special Health Care Needs: Methods and Implications

**DOI:** 10.3390/nu16091274

**Published:** 2024-04-25

**Authors:** Ruby Natale, Michelle Schladant, Martha H. Bloyer, Julieta Hernandez, Joanne Palenzuela, Yaray Agosto, Youmeizi Peng, Sarah E. Messiah

**Affiliations:** 1 Mailman Center for Child Development, Department of Pediatrics, University of Miami School of Medicine, Miami, FL 33136, USA; mschladant@med.miami.edu (M.S.); jhernand@med.miami.edu (J.H.); jpalenzuela@miami.edu (J.P.); yagosto@med.miami.edu (Y.A.);; 2 Department of Physical Therapy, University of Miami, Coral Gables, FL 33136, USA; mhb79@med.miami.edu; 3 University of Texas Health Science Center at Houston School of Public Health, Dallas, TX 75207, USA; sarah.e.messiah@uth.tmc.edu; 4 Center for Pediatric Population Health, University of Texas Health Science Center at Houston School of Public Health, Dallas, TX 75207, USA; 5 Department of Pediatrics, McGovern Medical School, Houston, TX 77030, USA

**Keywords:** children, special health care needs, obesity, prevention, early childhood

## Abstract

Children with disabilities have higher prevalence estimates of obesity than typically developing children. The Healthy Caregivers–Healthy Children Phase 3 (HC3) project implemented an obesity prevention program adapted for children with special health care needs (CSHCN) that includes dietary intake and physical activity (PA) components. The primary outcome was a change in dietary intake, PA, and the body mass index (BMI) percentile. Ten childcare centers (CCCs) serving low-resource families with ≥30 2- to 5-year-olds attending were randomized to either the intervention (n = 5) or control (n = 5). The HC3 CCCs received (1) snack, beverage, PA, and screen time policies via weekly technical assistance; (2) adapted lesson plans for CSHCN; and (3) parent curricula. The control CCCs received a behavioral health attention curriculum. HC3 was delivered over three school years, with data collected at five different timepoints. It was delivered weekly for six months in year one. To ensure capacity building, the HC3 tasks were scaled back, with quarterly intervention delivery in year 2 and annually in year 3. Adaptations were made to the curriculum to ensure appropriate access for CSHCN. Given that the program was being delivered during the COVID-19 pandemic, special modifications were made to follow CDC safety standards. The primary outcome measures included the Environment and Policy Assessment and Observation (EPAO) tool, standardized dietary intake and PA assessments, and the child BMI percentile. CCCs are an ideal setting for targeting CSHCN for obesity prevention efforts as they provide an opportunity to address modifiable risk factors.

## 1. Introduction

One in four children in the US under the age of 5 years have either overweight (body mass index [BMI] >85th–< 95th percentile for age and sex) or obesity (BMI > 95th % for age and sex) [1]. In 2017–2018, the obesity prevalence was 13.4% among 2- to 5-year-olds [2]. Children with special health care needs (CSHCN) and ethnic minority children are disproportionately affected by the obesity epidemic [2,3]. Moving forward, CSHCN will be collectively defined as those with developmental, physical, or intellectual disabilities, with special emphasis on those with developmental disabilities as specified by the Child and Adolescent Health Measurement Initiative (CAMHI) [4]. Children at the intersection of these two disparities (ethnic minority status and CSHCN) have rarely been studied, yet obesity is 38% higher in CSHCN compared to their peers without disabilities and three to four times higher in non-Hispanic Black (NHB) and Hispanic preschool age children compared to non-Hispanic Whites (NHWs) [2,4,5]. These statistics are concerning because preschool-age children with obesity are (1) five times more likely to have overweight during adolescence, (2) four times more likely to have obesity as adults compared to their normal weight counterparts [6,7], and (3) contrary to popular belief, children do not “grow out of their baby fat”. In fact, excessive weight gain in the first five years of life can alter developing neural, metabolic and behavioral systems in ways that increase the risk of chronic disease (type 2 diabetes, cardiovascular disease, hypertension, and stroke) later in life [8,9]. Reports have shown that childhood-onset obesity significantly contributes to increased morbidity and mortality in adulthood [10], particularly among ethnic minority groups, who are disproportionately affected by many of these chronic conditions [11]. Our team has reported that not only are US adolescents with disabilities significantly more likely to have overweight and obesity compared to their peers with no disabilities, they are over three times as likely to have elevated blood pressure, insulin, lipids and metabolic syndrome, the hallmark precursors to type 2 diabetes [12].

Limited research exists on the causes, consequences, prevention, and treatment of obesity among CSHCN. Virtually no research has addressed the intersection of CSHCN and obesity prevention in the early childhood years (ages 2 to 5 years) or the preschool/childcare setting. Yet, the first years of life may present the best opportunity for obesity prevention. During early childhood, lifestyle behaviors that promote obesity are formed, and it is easier to establish new healthy-weight-promoting behaviors than to change poor, existing ones [13]. Childcare settings offer a potentially powerful infrastructure to implement such efforts because: (1) 50% of CSHCN are enrolled in daily, out-of-home childcare [14]; (2) CSHCN from low-income backgrounds consume 50–100% of their Recommended Dietary Allowances in the childcare setting [15]; (3) many children spend the majority of their waking hours out of home and in the childcare setting [16]; and (4) access to high-quality nutrition is a major health disparity [17].

This study was designed to build on our previously designed Healthy Caregivers–Healthy Children Phase 1 and Phase 2 programs. The goal of HC2 Phase 1 was to test effective healthy weight strategies in early childhood settings utilizing educators and parents as nutritional and physical activity gatekeepers and healthy lifestyle role models for 2- to 5-year-olds [18]. We developed and delivered a bilingual (English/Spanish) toolkit to increase dietary intakes of fruits and vegetables; decrease dietary intakes of foods high in solid fats and added sugars; decrease the amount of television viewing and computer use; increase physical activity in preschool children; and decrease the proportion of preschool children on an unhealthy growth trajectory [19]. The goal of HC2 Phase 2 was to test the effectiveness of implementing the HC2 curriculum via a Train-the-Trainer design [20]. Overall, the findings across both phases showed that our HC2 obesity prevention intervention (1) is effective in maintaining a healthy weight and decreasing the obesity prevalence in ethnically diverse preschoolers; (2) significantly improves the consumption of healthy foods, including fruits and vegetables; (3) significantly decreases the consumption of unhealthy foods; and (4) increases physical activity among children and their families [18]. In a sub-sample of CSHCN, we showed similar, promising results that support the expansion of HC2 among this group specifically [21,22]. Therefore, the primary Aim 1 of this Phase 3 Healthy Caregivers–Healthy Children (HC3) program was to adapt and evaluate our HC2 program to specifically include childcare centers serving low-income, multiethnic CSHCN. The secondary Aim 2 was to determine the mediators that could impact obesity-related outcomes within this population.

## 2. Materials and Methods

### 2.1. Study Design Overview

This 3-year randomized controlled cluster trial was conducted with 10 childcare centers—5 intervention and 5 control (ClinicalTrials.gov ID: NCT05106426). The measures were completed over the following 5 timepoints (T): T1 at the beginning of school year 1, or pre-intervention (Time 1 = baseline, October–December 2021); T2 at the completion of post-intervention (Time 2 = end of the first school year, April–June 2022); T3 at the beginning of the second school year (September 2022); T4 at the end of the second school year (April–June 2023); and T5 at the beginning of the third school year (October–December 2023).

### 2.2. Study Participants and Inclusion/Exclusion Criteria

**Figure 1:**
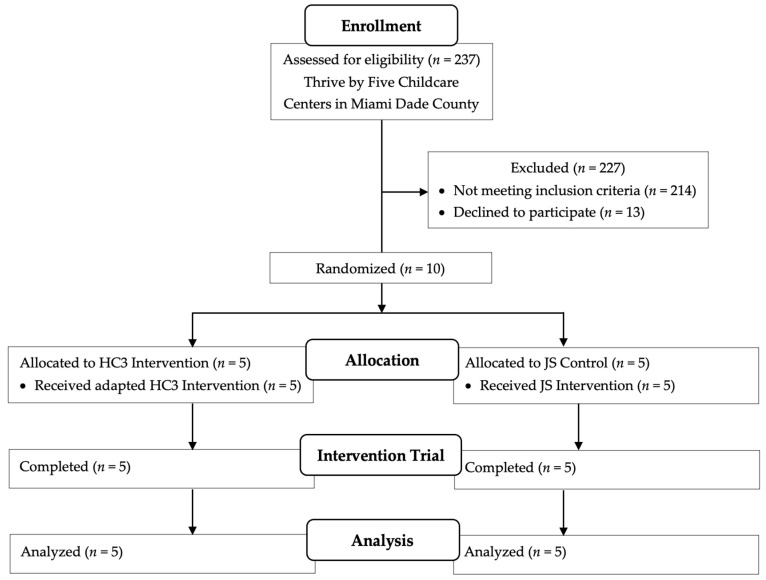
CONSORT flow diagram.

Childcare centers must have met the following criteria to be included in this study: (1) ≥30 children ages 2- to 5-years-old enrolled; (2) be located in a low-income census tract zone; (3) at least 50% of the children are ethnic minorities; (4) at least 10 children in the center with a disability or at risk of delays as per an evaluation; and (5) center directors and teachers agree to participate. Childcare centers were excluded if they did not meet these criteria (see Figure 1). Children were included if a parent/guardian consented to study participation. Children with feeding tubes, failure to thrive or children who bring their own meals due to dietary restrictions were excluded. It is likely that children with extreme allergies are not participating in group childcare settings as center directors indicate a very low number of children with any allergies or sensitivities at this time. Children identified by parents as failure to thrive (<5th BMI %ile) on the demographic form were excluded.

### 2.3. Recruitment

Centers that were eligible were contacted by the program coordinator via telephone, and after being given a description of HC3, were asked to provide a verbal commitment of interest in participating. Study staff followed up with those centers that were interested in participating to obtain a signed consent form. Once a signed consent was obtained, study staff met with the center director to discuss the role their center would have in the study. Parents/guardians were recruited for study participation via verbal announcements, informational flyers, and letters sent home. Family members who returned a signed interest form were contacted by telephone. After verbal consent was obtained, an appointment was made for the child and parent to be seen at the childcare center, at which time the written informed consent from the parents was obtained and initial baseline data collected. Due to COVID-19, appointments had to be held outside of the center or indoors in an area designated by each center for parent drop off and pick up and in line with CDC guidelines on social distancing. Parents were also given an option to complete paperwork via phone or electronically through a secured REDCap server after obtaining a signed consent. In addition to following the childcare centers’ COVID-19 protocol, the research assistant also had to follow the university’s COVID-19 protocol, which was consistent with CDC guidelines and updated as new stipulations were released [23].

Primary caregivers were given a USD 20 gift card for completing the nutritional and physical activity assessment battery for their child. The same compensation schedule was used for the intervention and control participants. Incentives were issued for completion of pre-measures (Time 1 = baseline, October 2021) and at completion of all post-measures. We increased the incentives over the course of the study to assist with longitudinal retention. This compensation amount was appropriate, given that it took approximately 30 min to complete the assessment battery.

### 2.4. Measures

#### 2.4.1. Intake Interview

Our HC2 baseline questionnaire was used to capture age, sex, and race/ethnicity (children and parents). Given the ethnically diverse target population, cultural influences were explored as possible moderators impacting HC2 adaption, such as language(s) spoken in the home and level of acculturation using the Stephenson Multigroup Acculturation Scale [24]. The Collins Figural Rating Scale [25], a common pictorial tool for measuring body figures in preschool children, was used to assess parent perception of child anthropometric phenotype status. Caregivers were asked to identify their child’s phenotype from the seven figures on the silhouette chart featuring preschool-aged figures. Images 1–3 are categorized as normal weight and images 4–7 are categorized as unhealthy weight [26]. Lastly, caregivers were asked to identify their child’s disability status and developmental milestones.

#### 2.4.2. Child Food Intake

The child’s nutrition intake was measured by the (1) Healthy Kids Checklist (HKC) [27,28] and (2) Food Frequency Questionnaire (FFQ) [29]. The HKC is a parent-reported measure of child dietary intake at home of fat, fiber, fruit/vegetable, calcium/dairy, and sweetened beverages, as well as nutrition behaviors, including eating in restaurants, breakfast skipping, and physical activity habits, and parent role-modeling of healthy eating. The HKC uses visuals with text at a second-grade reading level and is validated for our target age population [27,28].

The FFQ measures nutritional behaviors at school based on center director reports of menu items served [29]. We have used this measure previously and modified it to include categories of food items with the frequency served per week (i.e., juice, 1% milk, fresh fruit etc.) [18,20].

#### 2.4.3. Caregiver Nutrition and Physical Activity

The Fruit and Vegetable Inventory (13 psychosocial items) measures fruit and vegetable (FV) intake and perceived control, self-efficacy for eating healthier, readiness to eat more FV, and perceived diet quality [30]. It also measures role-modeling variables such as what items are consumed when their children are present. 

The Physical Activity Self-Efficacy Scale is a 5-item reliable questionnaire that measures confidence regarding participation in physical activity (e.g., addresses overcoming barriers to becoming or being physically active) and role-modeling [31]. 

#### 2.4.4. Anthropometric Variables

Height (stadiometer) and weight (digital scale) were collected for each child and converted to an age- and sex-adjusted BMI percentile (study primary outcome). The data collection methods were based on US Department of Health and Human Services (US HHS) guidelines [32]. 

#### 2.4.5. The Health Environment Rating Scale

The Health Environment Rating Scale (HERS) is an observational measure that examines children’s levels of exposure to time spent in front of screens (sedentary behavior) and in physical activity, as well as the quality of food and beverage types served in the classroom [33]. Classrooms were observed for a 6 h period and were rated on 21 items that compose the 4 scales (screen time, physical activity time, beverage type, quality nutrition intake). Items were rated on a Likert scale from 1–7 (1 sub-optimal to 7 above optimal). Trained research assistants were required to reach 80% interrater reliability prior to conducting observations on their own.

#### 2.4.6. The Environmental and Policy Outcomes

The Environmental and Policy Outcomes (EPAO) tool assessed the level of implementation of the HC2 policies through direct observation of the diet and physical activity environments in the childcare facilities [34]. The EPAO required a one-day evaluation that began from the first meal served to the last child that left the facility. It is the first tool to measure critical components such as the type of food served to children, teachers and facility staff. The scales directly match the HC3 toolkit policies. This was also a primary study measure conducted by a trained research assistant who met the interrater reliability of 80%.

All the study measures apart from the intake interview were collected at all five timepoints.

## 3. Intervention

### 3.1. Detailed Description of HC2 Tiers

This program was modeled after our USDA-funded Phase 1 and Phase 2 work that developed an obesity prevention program for childcare centers [18,20]. A general description of the HC2 toolkit can be found elsewhere [18,19]. In brief, the HC2 toolkit includes 42 lesson plans for teachers to use that show them a script for implementation and how each lesson plan meets the state of Florida classroom curriculum standards as defined by the Classroom Assessment Scoring System^®^ (CLASS) [35]. In this current Phase 3, we have included recommended modifications for CSHCN. The HC2 toolkit consists of material designed to incorporate all the current nutrition and physical activity policy requirements for preschool children in Florida and embraces best-practice guidelines from the American Academy of Pediatrics Caring for Children, 3rd Edition [36], Let’s Move [37], and USDA Team Nutrition [38,39] as well as UM-developed policies guided by evidence-based research [18,19]. These policies are the foundation of the entire program and include: (1) Snack Policy—fresh fruits and vegetables and whole grains (no sweets, high-fat foods) are the snack foods of choice/preferred foods; (2) Beverage Policy—serve low-fat (1%) or nonfat milk only (no whole milk), serve juice only one time per week, make water available all day and encourage it as the beverage of choice; (3) Physical Activity Policy—at least 90 min of physical activity every day; and (4) Screen Time Policy—limits screen time to 30 min per week, which does not include screens used for educational purposes or for communication (i.e., ACC device, tablet, or smart board). Recommendations were made to the centers, and UM coaches provided weekly Technical Assistance visits to assist with toolkit implementation and address barriers. In addition, five parent and teacher role-modeling workshops were held that focused on one of the four policies and one on the adaptations for CSCHN. Both the parent and teacher curriculums mirror key concepts taught to the children so that caregivers can role model and reinforce topics their children receive in the classroom about healthy lifestyle choices. All the parent and teacher workshops were held at the childcare centers and delivered by the Curriculum Specialist. The toolkit is available in English and Spanish.

### 3.2. Adaptation of HC2 for CSHCN

Each month one of the policies was the focus of the intervention and thus adaptations were made to the corresponding lesson plans to accommodate CSHCN on a monthly basis. A team consisting of a dietitian, special educator, physical therapist, and pediatric psychologist met monthly to adapt the lesson plans. Table 1 provides a summary of the modifications that were made to the HC3 curriculum based on the disability type and for the four program policies. For example, children with intellectual disabilities may need support in self-help skills, problem solving, and learning; children with physical disabilities may need support to address fine and gross motor skills; and children with ASD may need support in behavior, social skills, and communication. For children with more complex special needs, a physical therapist and a special educator were available to further modify the curriculum based on the child’s individual education plan (IEP).

The curriculum included 42 lesson plans, and each lesson plan had its own page of the general adaptations that could be utilized if that classroom had CSHCN. Adaptations to the curriculum were based on recommended practices by the Division for Early Childhood (DEC), which are evidenced-based strategies to support the needs of young children with disabilities [42]. DEC Recommended Practices emphasize the use of assistive technology adaptations and strategies to promote access to and participation in early learning experiences [42]. As shown in Table 2, suggestions for six different adaptations were utilized that included considerations for fine motor, gross motor, visual, social-emotional, communication, and participation challenges. A variety of assistive technology tools, such as visual supports, adapted books, switch-activated toys, visual timer, and book stands, were included to accommodate a wide range of abilities. Not all the adaptations were needed for all the lesson plans. Depending on the lesson plan, anywhere from one to all six adaptations may have been necessary.

Table 3 demonstrates a sample lesson plan and how the adaptations for CSHCN are embedded into the curriculum. For this lesson plan, which focused on physical activity by having children participate in yoga, adaptations were needed that included the use of visual aids, communication aides, and gross motor aides. The study team was available to make further accommodations for individual students based on their IEP.

### 3.3. Adaptations Based on the COVID-19 Pandemic

Given that COVID-19 posed many challenges for childcare centers, the curriculum was adapted to offer a telehealth delivery option. Depending on the variant spikes in Miami-Dade County, the centers limited the amount of in-person services they would allow in their centers due to the risk of transmission of the virus. The curriculum began in January 2022 during a spike of the Omicron variant and so for the month of January services had to be implemented virtually. The childcare centers used an iPad and were taught how to use Zoom for business. A Zoom account was set up for all the centers that did not already have one. Weekly consultations were held virtually with teachers during lunch time or breaks. Parent workshops were held virtually one time per month, in which all participating parents from all five centers were invited to participate. As COVID-19 rates fluctuated and declined, the use of a hybrid model in which there were some in-person and some virtual workshops and consultations became an option.

### 3.4. Control Condition

Those centers randomized to the control arm received an attention control consisting of Jump Start, a behavioral support consultation program. Consultants virtually implemented the Jump Start program at the same level of exposure and contact time as the treatment arm. The control centers received all the same pre–post measures and incentives as the intervention arm (to ensure retention/reduce loss to follow up). The Jump Start program has been in existence for 4 years and has already been targeted for CSHCN, so it did not need to be adapted.

## 4. Analysis Plan

The analysis plan will include hierarchical linear (i.e., multilevel) growth modeling techniques (data are currently being analyzed). The cluster-randomized design of this study yields a so-called 3-level model, where repeated measures are nested within children (level 1), individual teachers and parents (level 2), and children, teachers and parents are nested within preschools/childcare centers (level 3). This basic modeling framework will be used for all the repeated measures outcomes (e.g., parent and teacher efficacy measures and child BMI). Moreover, the statistical model can be expanded to address the relevant moderating effects, and thus, it is an ideal statistical method to apply to our proposed study, given our exploratory approach in Aim 2 in particular. However, in terms of both study aims, we are specifically interested in what it is about the system (e.g., childcare center/preschool, home environment) that might influence a child’s BMI. A simple way to examine this is to explore other study measurements and see how they may vary between the treatment and control conditions. This is perhaps the simplest method and has also been called a “secondary outcomes” approach [43]. This may be regarded as a first step approach in “exploring” for possible moderators and/or mediators. As an example, if the teacher’s “healthy lifestyle role-modeling scores” are higher in the treatment than the control group, then it might be the case that modeling influenced the system (given that there was a treatment effect on BMI). However, one can quickly see that this approach can create many different explanations, which is why the approach is labeled “exploratory”. Specifically, in Aim 2, we can model the outcome (e.g., BMI) and then, along with the treatment classification, include other variables (e.g., ethnicity, level of parent and/or role modeling, agency adherence to curriculum) we predict may moderate the treatment effect. These other variables will primarily need to be measured at the subject (individual) level. The issues with nested effects only apply to methodology that involves the generation of *p* values (inferential stats), so variables measured at the teacher or family level could be included here.

### Power Analysis

Within the cost and time limitations of the current grant, the maximum number of schools in which a designed randomized trial is possible is 10 (5 CCC in each arm; intervention versus control). The CCC is the primary unit of analysis as this is the level in which the intervention will be applied. Past research has shown that the intraclass correlation (i.e., within school dependency of students within a school) is, in general, very low and for all practical purposes close to zero [44]. Given the standard deviation of the student-to-student BMI z scores is by definition 1.0, and it is expected that, on average, the number of students per school will be approximately 30 (accounting for a 10% attrition rate) the school-to-school variation (i.e., standard deviation) should be approximately 0.1690 standard deviation units. Given these estimates, we determined how much of a statistical difference in the BMI z scores can be detected for a given power (i.e., 0.80) using a Type I error rate of 5% (two-tailed). Assuming that the correlation of the pre to post BMI z scores is fairly high (i.e., 0.80; which is roughly equivalent to the reliability coefficient of the BMI), the standard deviation of the difference in the BMI z scores (i.e., post–pre) works out to approximately 0.1070. The detectable effect size equates to a Cohen’s d statistic of 0.20. According to Cohen’s taxonomy, this would be considered a “small” effect with a corresponding R^2^ (i.e., percent variance explained of 1%) [44]. 

## 5. Discussion

While numerous studies have found the prevalence of obesity is higher among CSHCN than in the general population, limited research exists on the contributing factors, consequences, prevention, and treatment of overweight and obesity among CSHCN, especially very young children. To compound this issue, even at young age, children who are from ethnic minorities with a disability are disproportionately affected [45]. Article 25 of the Convention on the Rights of Persons with Disabilities states that persons with disabilities not be withheld from access to mainstream health promotion programs [46]. We are addressing this critical gap, given there is limited research on interventions that target CSHCN in childcare settings, particularly among those from ethnic minority groups [47].

### 5.1. Barriers to and Facilitators of Implementation

Addressing the barriers faced by children with special healthcare needs (CSHCN) in participating in health and wellness programs requires a multifaceted approach. One significant barrier is the lack of teacher training and awareness on how to include CSHCN in such programs, alongside limited access to information and resources. Simple modifications based on identified disabilities, as outlined in Table 1; Table 2, offer potential solutions. Additionally, adapting the curriculum to ensure inclusivity and providing support to teachers to address implementation barriers are crucial elements of the weekly technical assistance. By implementing such programs, we can enhance our understanding of the obesity risk factors in CSHCN, thus bolstering the evidence base for wellness-promoting policies in childcare centers. Effective delivery strategies in targeted preschools can mitigate health disparities and improve the well-being of early childhood populations, which is particularly important in reducing obesity rates. Furthermore, amidst the ongoing challenges posed by COVID-19, childcare centers must adapt interventions to comply with CDC guidelines on social distancing and mask usage. While these guidelines have hindered in-person interventions like HC3, virtual modifications have shown promise in offering adaptable curriculum and equipment, thus ensuring continued support for CSHCN in maintaining healthy weights despite the pandemic’s constraints.

### 5.2. Childcare Centers and Role-Modeling as a Medium for Change

Childcare centers are an ideal setting for targeting this population because they provide an opportunity to modify most risk factors for childhood-onset obesity, including increased physical activity, provision of healthy nutrition, and healthy lifestyle education. A program such as ours that targets younger children is likely to be more successful because eating patterns are less rigid earlier in a child’s life. Furthermore, we include the family and home environment, given the importance that parents/caregivers play in shaping the dietary patterns of children. If home and parent/caregiver practices are unhealthy, it can put young children at risk of obesity because they depend on adults for their nutritional needs [48]. Young children learn by observing the behavior of the important adults in their lives, and if their parent/caregiver enjoys a particular food, a young child is likely to view that food as a safe and nutritious choice to try for themselves. Parents/caregivers have an impact on the nutritional habits of the children under their care, not only by making choices regarding the types of foods that are available but also by influencing children’s attitudes and beliefs about those foods. The USDA’s concept of the “nutritional gatekeeper” suggests that the person buying and serving the food, which is most often a parent/caregiver, has a powerful impact on the child’s outcomes [48]. Our multi-tiered design with opportunities for caregiver training and technical assistance will further address the needs of children with disabilities by acknowledging the other environmental and behavioral factors that parents/caregivers may face in serving healthy meals (stress of raising a child with CSHCN, picky eaters) and encouraging physical activity (need for increased supervision, additional opportunities to participate) [49,50,51]. 

## 6. Conclusions

Efforts to prevent obesity and overweight during early childhood among CSHCN have been scarce. Yet, supporting a healthy weight gain trajectory, quality nutritional intake and habits, and sedentary behavior would have substantial benefits for CSHCN, their families, and society at large. Examining the policies in the childcare setting specifically is needed, as this setting could be especially strategic for effective healthy weight development interventions. Our methods are replicable and scalable for testing the effects of obesity prevention programs in childcare centers and have the potential to improve early childhood nutrition and physical activity for those with special health care needs. 

## Figures and Tables

**Table 1:** Modification of the curriculum based on the policy and disability eligibility category of the child’s IEP.

Disability Type	Beverage/Snack Policy	Screen Time/Physical Activity
Intellectual Disabilities	Learn to identify fruits and vegetables using real foods. Tasting and using pictures (happy face, neutral, unhappy) to determine the child’s acceptance of liking of the food. Provide picture-supported guides (e.g., line drawings, photographs). The National Center on Physical Activity and Disability suggests parents include children in food preparation, washing vegetables, setting table [40]. Caregivers will be encouraged to recognize and honor their children’s satiety cues. Children should be allowed to eat slowly and without distraction.	Include peer-modeling in sports. Use traditional games: hopscotch, jumping rope, kicking, catching, bats, balls, tricycles. Set up a repetitive routine that will allow them the opportunity to master skills. Start with small activity goals and build on them over time. Use structured exercises, and sports and keep PA simple, like running, jumping. Parents will be encouraged to choose an activity the child enjoys—music and dance are popular [41].
Orthopedic Impairments	Our physical therapist can recommend a position for feeding that supports the child, minimizes abnormal tone and reflexes, and makes it easier to swallow food. Children with cerebral palsy often consume less food due to tongue thrust, poor lip closure, or abnormal reflexes. Our team nutritionist, physical therapist and pediatrician will provide recommendations for caregivers to assist with prolonged mealtimes or unfinished meals. Children will be offered foods with different food textures. Some foods can be smooth and easily eaten. Other foods should have more texture (with lumps or in pieces) to encourage tongue movements or chewing. Our dietician specializing in feeding can determine what food textures are appropriate for a child. Children will be encouraged to participate in the nutrition lesson plans using assistive technology devices such as adaptive books on learning about the body.	Provide playground adaptations to ensure children can participate in the recommended amount of PA. Use adaptive switch so child can move swings Provide adapted bicycles for movement for those in wheelchairs, adult-led activities in structured settings. Coaches will be present and serve as an aid while utilizing adaptations. Include peer-modeling in sports. Use Nintendo Wii exercise game. Purchase big instruments (oversized tambourine, cymbals, large bells they can push) for the playground to encourage music and movement.
Autism Spectrum Disability (ASD)	Efforts will be made to encourage children to try new fresh fruits and vegetables that can be served at snack. The team PT, speech, dietitian, pediatrics will be available to provide alternatives based on texture and food selectivity to accommodate a child’s preferences. Touching and smelling may be the first step in trying new foods.	Given screen time is respite time for caregivers and can be used for calming time for some children, alternate soothing techniques will be incorporated (i.e., playing music, rocking/swinging) [21]. Create a vegetable/butterfly garden to assist with building up the middle of the playground and prevent isolation by going to the corners of the playground. Sensory table/water play for those with sensitivities.

**Table 2:** List of adaptation materials based on types of special needs.

Disability Type	Beverage/Snack Policy
Fine Motor Challenges	Popsicle sticks will be available to assist children who experience difficulty grasping, holding or using fine motor skills. Page fluffers represent a simple adaptation made for books or other reading material that makes pages more accessible to turn. Fluffers (popsicle sticks) will be provided for all the books provided. Adapted scissors are provided for students to participate in art activities. This will allow children access and coordinate scissors to cut out a variety of cut outs or material shapes.
Gross Motor Challenges	A binder is provided to be used as a reading stand or easel for children requiring additional assistance during activities: circle time, group, or individually. The binder can help display tabletop materials that involve reading or writing tasks. This can help with posture and present material closer to the child’s eye level.
Visual Aids	A 12-month subscription to the Lesson Pix website is provided. This allows for teachers, and staff, to design, create and adapt materials according to the lesson plan activities and to the children’s individual needs.
Social-emotional	The Kadam Visual Timer is available to assist with organization and concentration. This timer can be used to set time limits or ease transitions between tasks during lessons or other teacher planner activities.
Communication	The iTalk2 communication tool is provided for lesson activities to enhance the participation of students with limited verbal speech. The use of iTalk2 has been incorporated in the various lesson plans to promote child participation and engagement in the classroom activities. The AbleNet5” Big Red Twist Switch is an adaption that allows children with disabilities to access activities and lessons with the slightest touch of a button. The Switch can be used with any accessible toy, like the bubble machines included.
Participation	Two types of bubble machines are adapted with the switch-adapted toy to enhance and foster the abilities of children who are unable to interact with conventional toys or participate in lesson activities.

**Table 3:** Sample lesson plan for the HC3 physical activity curriculum policy.

Animal Dice Lesson Plan			
**Curriculum Policy: Physical Activity** Age Level: 2–5 Lesson Duration: As needed Material: Animal dice 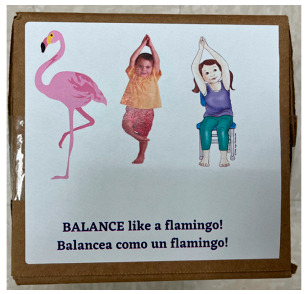	**Focus of the Lesson:** Getting children to move their bodies in new and creative ways It is important for teachers to model (ex. balancing when the dice says balance). New Vocabulary: March, fly, balance, and gallop CLASS Associations: Positive climate, teacher sensitivity, and behavior management	**Note:** If children have difficulty performing the exact movement described, teacher should give praise for trying and for moving their bodies, instead of correcting them. Children with difficulties balancing on one foot can hold onto a chair or put one hand on the wall to help them. Children who have difficulty hopping on one foot can jump on two.	
**Before the Lesson**	**During the Lesson**	**After the Lesson**	
Teacher will read the instructions below prior to the activity. Practice the activity. Practice new vocabulary words to emphasize the lesson.	Review behavior expectations Introduce the activity to children Teacher will model the activity with enthusiasm, using specific praise and providing additional assistance to those needing it.	Encourage children to have something to drink (ex. water).	
**Special Need Support**			
**1. Visual Aide**: Visuals of different types of animals and children’s movements provided on the dice to facilitate students’ participation in the activity. Additional visual aids with adaptations to the physical movements have been provided on the dice.	**2. Communication Aide**: A communication tool will be provided for this lesson to enhance the participation of students with limited verbal speech. For example, students may use the iTalk2 to request help, to request for turns, and to express “I like it” or “I do not like it” to comment on the activity.	**3. Gross Motor Aide**: Additional visuals with children using different poses as adaptations to the physical movements have been provided on the dice for children with limited motor skills. Assistance can be provided by teacher, staff, or peer either through direct or limited support.	

## Data Availability

The original contributions presented in the study are included in the article, further inquiries can be directed to the corresponding author.
